# Complete genome sequence of *Paenibacillus* spp. strain AASFL403 isolated from *Astracantha arnacantha*

**DOI:** 10.1128/mra.00222-25

**Published:** 2025-07-31

**Authors:** Yancho Zarev, Preslav Enchev, Iliana Ionkova, Ivan Stoikov, Deyan Donchev, Ivan N. Ivanov

**Affiliations:** 1 Faculty of Pharmacy, Medical University of Sofia 58789, Sofia, Bulgaria; 2 Department of Microbiology, National Center of Infectious and Parasitic Diseases 249307, Sofia, Bulgaria; University of Maryland School of Medicine, Baltimore, Maryland, USA

**Keywords:** *Paenibacillus*, endophyte, *Astragalus*, genome

## Abstract

We present the complete genome sequence of *Paenibacillus* spp. strain AASFL403, an endophyte from *Astracantha arnacantha*, a Bulgarian endemic plant. Members of the genus *Paenibacillus* are distinguished by their abilities to promote plant growth, function as biocontrol agents, and facilitate bioremediation.

## ANNOUNCEMENT

*Paenibacillus* is a genus of Gram-positive, motile, rod-shaped bacteria distinguished within the family *Paenibacillaceae* (1). Many species are associated with plant growth promotion and soil health. *Paenibacillus spp.* are known for their ability to fix atmospheric nitrogen, solubilize phosphate and potassium, produce phytohormones like indole-3-acetic acid (IAA), and act as biocontrol agents against plant pathogens. These bacteria are also valuable for bioremediation under adverse environmental conditions (1, 2). At the same time, *Astragalus* plant species are known for producing important bioactive metabolites (3). Some species, like *Astracantha arnacantha* (M. Bieb.) Podlech subsp. *aitosensis* (Ivan.), classified as a Tertiary relic, are endemic. According to recent advances, some important metabolites were described as products of the plant microbiome (4–6). Thus, understanding the diversity of *Astragalus* microbiome and studying the endophyte-host interactions could offer opportunities to mitigate economic losses in agriculture and explore their potential for biotechnological and medicinal applications (7–13).

We present the complete genome sequence of *Paenibacillus* spp. strain AASFL403, isolated as an endophyte from spring flowers of A. aitosensis, collected with the permission of the Bulgarian Ministry of Environment and Water (license no. 949/18.08.2022) from natural habitat (Lat. 42.726660, Lon. 27.279542), and maintained under standard laboratory conditions. The disinfection and isolation procedures were carried out following the methodology described previously (14). Briefly, the leaves underwent surface sterilization using the following sequence: immersion in 70% ethanol for 1 minute, treatment with sodium hypochlorite solution (containing 2.5% Cl⁻) for 4 minutes, exposure to 70% ethanol for 30 seconds, and three rinses with sterile distilled water. The sterilized material was placed on Man, Rogosa, and Sharpe (MRS) agar and incubated at 25°C, followed by three successive iterations of subculturing to ensure axenic culture establishment.

**Fig 1:**
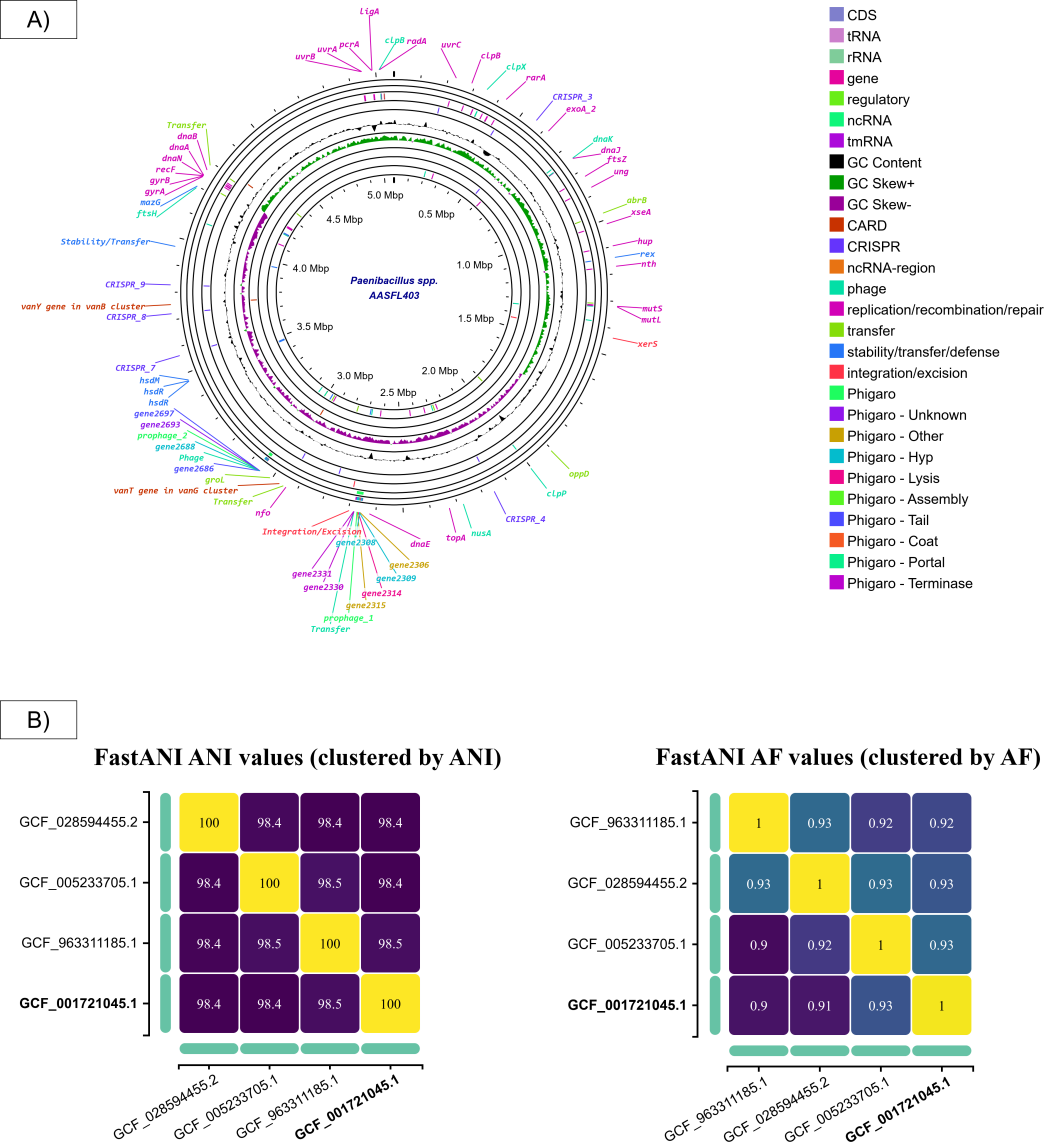
(A) Annotated genome map visualized in Proksee (https://proksee.ca/); (**B**) ANI and alignment fraction (AF) heatmaps of *Paenibacillus* spp. AASFL403 (GCF_963311185.1) and three closely related genomes of *P. nuruki* TI45-13ar (GCF_001721045.1), *Paenibacillus sp*. CFBP13512 (GCF_005233705.1), and *Paenibacillus sp*. PK4536 (GCF_028594455.2) from the GTDB.

Genomic DNA was isolated from a culture grown overnight at 30°C on Columbia agar using the Purelink Genomic DNA minikit (Thermo, USA) according to the manufacturer’s instructions. Long-read sequencing library was prepared from 600 ng gDNA with the Rapid Barcoding kit (SQK-RBK110.96), and sequencing was carried out on MinION Mk1C with v9.4.1 flowcell (Oxford Nanopore Technologies, UK). Super-high accuracy basecalling was performed with Guppy v6.4.8, whereas read quality and metrics were assessed with NanoPlot v1.29.1. Assembly and circularization were performed with the Trycycler pipeline v0.5.4 followed by long-read polishing with Medaka v1.8.1 (15). Post-assembly rotation was not performed. The resulting complete circular contig was further assessed with Quast v5.2.0 (https://github.com/ablab/quast) and CheckM v1.2.2. (https://github.com/Ecogenomics/CheckM) (Table 1). Default parameters were used except where otherwise noted. The NCBI Prokaryotic Genome Annotation Pipeline (PGAP) annotation was performed after the NCBI submission and additional features were retrieved with the Proksee webserver for illustration purposes only (Fig. 1) (16). Furthermore, the Genome Taxonomy Database (GTDB)-Tk tool v2.4.1 (17) analysis against the Genome Taxonomy Database (GTDB) R214 revealed only three closely related genomes with >95% average nucleotide identity (ANI), namely *P. nuruki* TI45-13ar (GCF_001721045.1), *Paenibacillus sp*. CFBP13512 (GCF_005233705.1), and *Paenibacillus sp*. PK4536 (GCF_028594455.2) (Fig. 1B).

**TABLE 1:** Genome sequencing metrics (NCBI RefSeq)

Genome statistics	*Paenibacillus* sp. (nuruki)
Isolate	AASFL403
Accession no.	NZ_OY725677
BioProject ID	PRJEB65278
Total reads (number/ Mbp)	71,734/385.5 Mbp
Read N50 (bp)	7,651
No. of contigs	1
Coverage	×76
Genome assembly size (bp)	5,066,866
CheckM completeness (%)	99.45
No. of CDSs^*a*^	4,394
G + C content (%)	39.48
No. of rRNAs	25
No. of tRNAs	77

^*a*^ CDS = Coding sequence.

## Data Availability

The Nanopore raw reads were deposited in ENA (ERR14387277) while the annotated assembly (NZ_OY725677) is available in Refseq NCBI.
